# Supplementary material for: Consensus label propagation with graph convolutional networks for single-cell RNA sequencing cell type annotation
Source: Bioinformatics. 2023 Jun 2;39(6):btad360. doi: 10.1093/bioinformatics/btad360 (PMC10272704; doi:10.1093/bioinformatics/btad360)
Supplement: btad360_Supplementary_Data [file btad360_supplementary_data.zip › appendix.pdf]

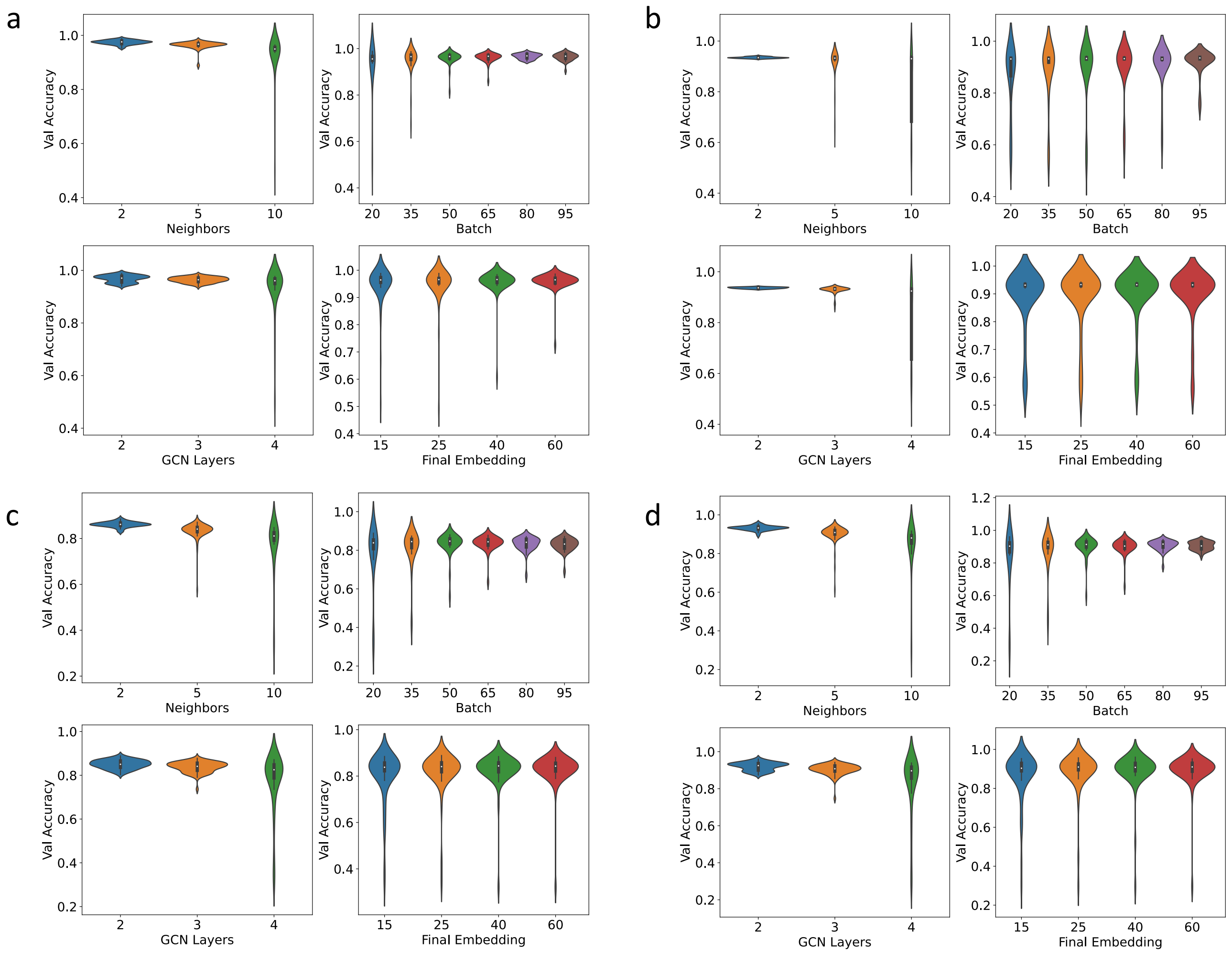

Fig. 1. Spread of validation accuracy scores as a function of various hyperparameters. The hyperparameters included are number of neighbors, batch size, GCN layers, and final embedding layer size. a. Testis data set. b. PBMC data set. c. Simulation 0.7 data set. d. Simulation 0.8 data set

A Hyperparameter Search Details

We performed a hyperparameter search over the number of neighbors k, batch size b, number of GCN layers l, and final embedding layer size e. Specifically, we tested number of neighbors at 2, 5, and 10, batch size at 20, 35, 50, 65, 80, and 95, GCN layers from 2 to 4, and final embedding sizes of 15, 25, 40, and 60. We tested GCN models on a validation set, consisting of 20 percent of the confidently labeled cells, for each combination of these hyperparameters. Figure 1 shows the validation accuracy distributions at each hyperparameter in each data set. Generally, we see high validation accuracy with lower neighbors and two or three GCN layers. Batch size and final embedding size seem to have a wide distribution of validation accuracy. For each data set, the combination of hyperparameters with the highest validation accuracy was selected for testing.

Our model architecture consists of l EdgeConv layers. Each EdgeConv layer consists of one round of message passing along edges of the graph, followed by a dense neural network model that maps from one layer's embedding space to the next layer's. Each node aggregates information using the sum of its received messages (from neighbors and itself). In all of our model architectures, the first layer takes input embedding size 500 and outputs embedding size 1000. The middle layers accept embedding size 1000 and output embeddings of the same size. The final layer accepts embedding size 1000 and outputs final embedding size e. Both hyperparameters number of layers l and final embedding size e are included in the hyperparameter search.

B de.facScale Simulation Parameter

See Figure 2 for details on how the de.facScale parameter affects classification difficulty. With de.facScale ≤ 0.5, the generated data is too difficult for any of the component tools to analyze – all of the component tools do poorly. On the other hand, de.facScale ≥ 0.9 is too easy – the cell types are well-separated enough in gene space that all component methods are able to classify them correctly. We generated simulated data with de.facScale values of 0.7 and 0.8, as these values produce a challenging, but still attainable, benchmark for classification.

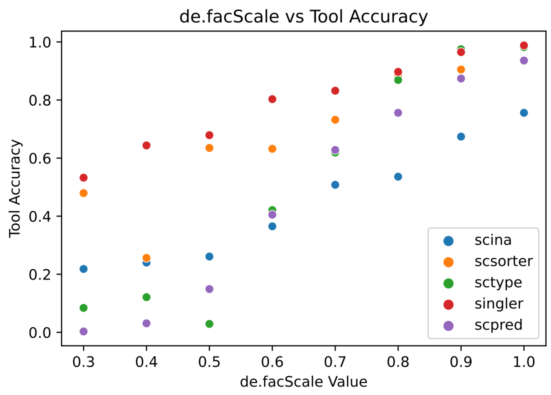

Fig. 2. Plot showing relationship between de.facScale simulation parameter and component tool accuracy. ScType, SCINA, ScSorter, SingleR, and ScPred were included as component tools.

C GCN Confusion Matrices

See Figure 3 for confusion matrices from GCN predictions on all data sets. We note that nearly all of these confusion matrices are characterized by a single cell type being the majority of unconfident cells. It is unclear why this is the case for the PBMC and testis data. In the synthetic data, one possible explanation is the way we choose marker genes. We randomly select five of the top ten differentially expressed genes in the simulated data of each cell type as markers (this is standard practice for using Splatter). It is possible this method results in some cell types with better markers than others. This was intentional, as in real-world data not all cell types will always have the same strength of cell type marker. For the actual data sets, this is likely because certain cell types (such as CD4 and CD8 T Cells) are more transcriptionally similar and likely to be misclassified. A common theme in both of these cases is that within each data set, some cell types are inherently easier to classify than others.

D Non-parametric Neighbor Majority Label Propagation

The results for the neighbor-majority label propagation approach across a variety of k values are shown in Figure 4. For each data set, we see that the convergence method does not greatly impact the accuracy for a given neighbor hyperparameter. We do see the neighbor hyperparameter is essential for the accuracy of this method.

E Testing in Simulated Data with Class Imbalance

To further test how our model performs in data sets with class imbalance, we generated two additional simulated data sets. These data sets were generated in the same way as the de.facScale 0.7 and 0.8 data sets, except proportions of 0.1, 0.2, 0.3, and 0.4 were used to generate the number of cells for each cell type. This resulted in 374, 318, 204, and 104 cells in Groups 1, 2, 3, and 4, respectively, in both data sets.

We then ran the same experiments as described in Experiment Settings and reported results in Table 1. Our model continues to out perform all underlying tools on overall accuracy in both data sets. Our GCN model also outperforms both the max consensus approach and the non-parametric neighbor majority approach.

These results in conjunction with the class imbalance seen in both the testis and PBMC data sets provide evidence for the robustness of our method in data sets with class imbalance.

F Testing in Simulated Data with Variation in Number of Marker Genes

To explore how varying the number of marker genes for each cell type affects both the marker-based component tools and our model, we created two more simulated data sets. These data sets were generated in exactly the same way the de.facScale 0.7 and 0.8 data sets were generated. We then ran all component tools with a full set of marker genes (five for each cell type) and with a subset of these marker genes. In the reduced marker gene set, Group 1 had only two marker genes, Group 2 had three, Group 3 had four, and Group 4 had all five marker genes. The non-parametric approach was not included as it is not directly relevant to testing how varying the number of marker genes affects the component tools and our model. Additionally, the same model hyperparameters used for the original simulated data sets were used for this experiment.

Tables 3 and 4 show the by cell type change in accuracy for each of the marker-based tools as a result of reducing the marker gene set. For scSorter, we see a large decrease in accuracy for Group 1, where the most marker genes were removed, for both data sets. For SCINA, we see very similar results with both sets of marker genes, except for Group 2 in the Simulation 0.7 de.facScale data set where there is a large increase in accuracy for Group 2. ScType accuracies remained the same across both gene sets and both data sets. Table 2 shows the accuracy results for both our Simulated 0.7 and 0.8 de.facScale data sets with all and reduced marker genes. In both data sets with reduced marker genes, we see decreased max consensus accuracy, indicating the tool consensus is worse when the marker genes are reduced. As a result, we see decreased accuracy in our model as well when the marker gene set is reduced.

Overall, the marker genes used do impact our model's performance. However, this impact is a result of decreased performance in the marker-based tools.

G Comparison Between DeepLIFT and Differentially Expressed Genes

To compare highly attributed DeepLIFT genes and genes found through differential expression analysis, we used the Seurat FindAllMarkers() function. We used default parameters except for only.pos, which we set to true. This function uses a Wilcox based differential expression test to return differentially expressed genes for each cell type. Cell types assigned by our GCN model were used for this analysis.

Table 5 shows the top ten highly attributed DeepLIFT genes and top ten positively differentially expressed genes for all cell types in the testis. In Spermatogonia, seven of the top ten highly attributed DeepLIFT genes are also identified by differential expression. Similarly, six of the top ten highly attributed DeepLIFT genes for Spermatocytes are shared with differential expression analysis. Finally, in Spermatids, only five of the top ten highly attributed DeepLIFT genes are shared.

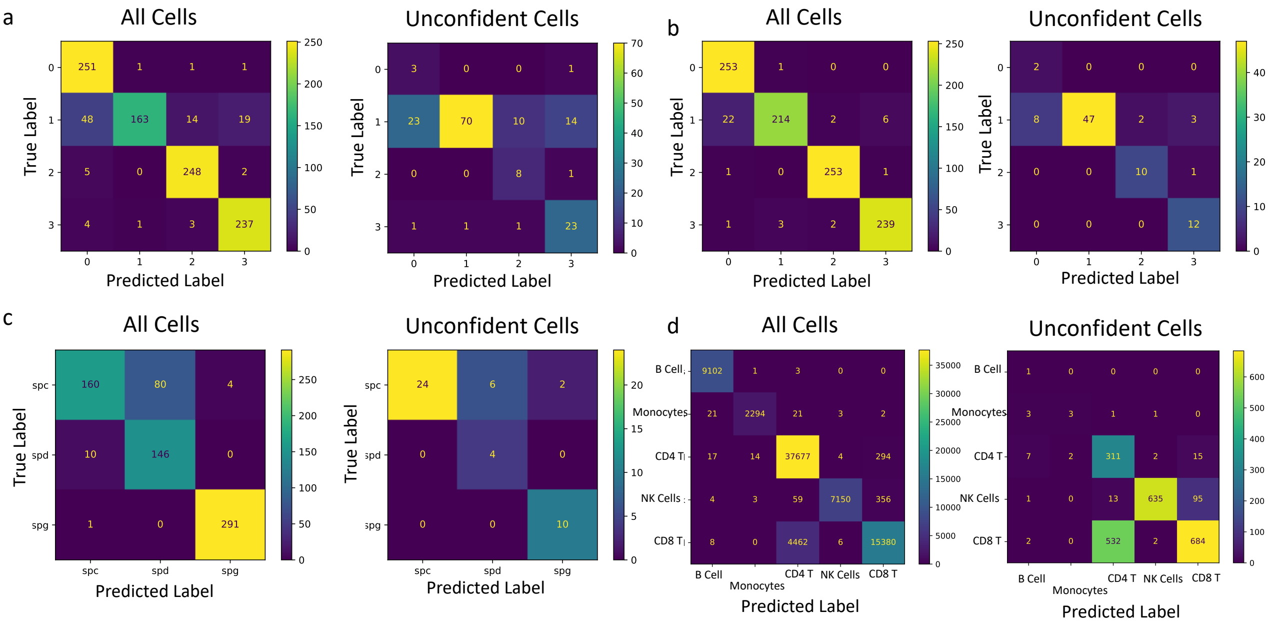

Fig. 3. Confusion matrices for GCN predictions in all and unconfidently labeled cells. a. Simulation 0.7 b. Simluation 0.8 c. Testis d. PBMC

Table 1. Accuracy scores (percentage of cells correctly classified) for simulated class imbalance data sets for both all cells and "unconfident cells": cells for which the underlying methods did not have consensus. GCN accuracies are the mean ± standard deviation of accuracies from five randomly initialized trials.

	Simulation 0.7 Imbalance		Simulation 0.8 Imbalance	
Method	All	Unconf. (70)	All	Unconf. (26)
Ours (GCN)	**94.0 ± .31**	62.0 ± 4.4	**99.5 ± .11**	**90.0 ± 4.4**
Max Consensus	91.8	30.0	98.1	34.6
Tool Avg.	78.8 ± 9.9	34.9 ± 28	88.2 ± 10	38.5 ± 32
ScType	75.3	25.7	95.4	42.3
scSorter	87.1	62.9	95.4	76.9
SCINA	70.4	10.0	74.9	3.8
SingleR	91.4	**65.7**	95.5	61.5
scPred	70.0	10.0	79.8	7.7
Non-Parametric	92.1	34.3	98.1	34.6

Table 6 shows the top ten highly attributed DeepLIFT genes and top ten positively differentially expressed genes for all cell types in the PBMC data set. For the top ten highly attributed genes in each cell type, seven, five, four, one, and three genes were shared with the top ten differentially expressed genes in B Cells, Monocytes, NK Cells, CD4 T Cells, and CD8 T Cells respectively. The low sharing between highly attributed and differentially expressed genes in T Cells likely stems from the low model accuracy identifying between CD4 and CD8 T Cells. We also found CD4 to be differentially expressed in CD4 T Cells after lowering the requirement for percentage of cells expressing the gene.

H Evaluation of Cell Type Similarities

To evaluate the difficulty of classifying our test data sets, we calculated the average distance between each cell type cluster for each data set. Figure 5 shows the results for this experiment in the form of heat maps. For each heat map, the scale goes from zero to the next highest integer above the highest average distance between any two cell types. This data shows that our simulated data sets consist of four cell types all with similar average distance from each other. One interesting aspect is that our Simulation 0.8 data set does not appear very different with this metric, despite being empirically easier to classify. In contrast to the simulated data sets, our testis and PBMC heat maps show varying similarities between cell type groups. For example. Spermatocytes and Spermatids have lower average distance than either of these cell types do with Spermatogonia. In the PBMC data set, CD4 and CD8 T Cells have lower average distance between each other than with other cell types.

In general, our simulated data sets show almost equal similarity between all cell types. In contrast, both real data

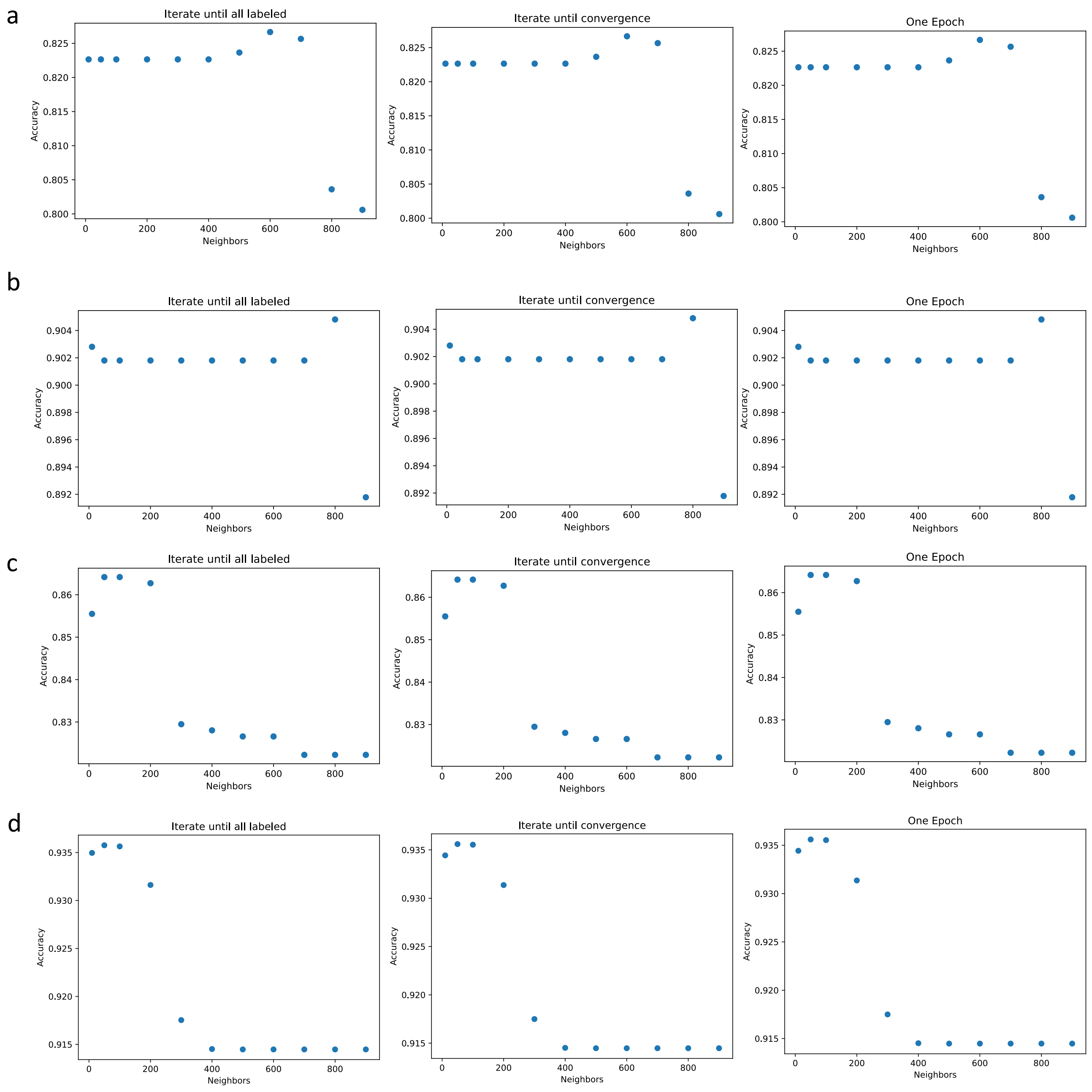

Fig. 4. Number for neighbors vs accuracy score for different convergence methods and data sets. a. Simulation 0.7 b. Simulation 0.8 c. Testis d. PBMC

sets show varying levels of similarity depending on which cell types are compared.

I Marker Genes Used

The gene markers for the testis data set were as follows:

- Spermatogonia: Dazl, Sycp1
- Spermatocytes: Insl6, Piwil1, Pttg1, Spag6, Mllt10, Aurka
- Spermatids: Acrv1, Spaca1, Tsga8, Tssk6

The gene markers for the PBMC data set were as follows:

- B Cells: CD19, MS4A1, CD79A
- Monocytes: CD14, FCGR1A, CD68, S100A12
- Natural Killer Cells: NCAM1, FCGR3A
- CD4 T Cell: CD4, CD3D, CD3E, CD3G
- CD8 T Cell: CD8A, CD8B, CD3D, CD3E, CD3G

Table 2. Accuracy scores (percentage of cells correctly classified) for simulated marker variation data sets for both all cells and "unconfident cells": cells for which the underlying methods did not have consensus. GCN accuracies are the mean ± standard deviation of accuracies from five randomly initialized trials. Simulation 0.7 and 0.8 All refer to the data sets where all five markers were used. Simulation 0.7 and 0.8 Reduced refer to the data sets where markers were reduced as described in Appendix F.

	Simulation 0.7 All		Simulation 0.7 Reduced		Simulation 0.8 All		Simulation 0.8 Reduced	
Method	All	Unconf. (148)	All	Unconf. (193)	All	Unconf. (50)	All	Unconf. (87)
Ours (GCN)	**96.2 ± .23**	**89.5 ± 1.6**	**91.8 ± .51**	**80.2 ± 2.4**	**98.8 ± .20**	**94.0 ± 4.0**	**98.1 ± .11**	**92.9 ± 1.3**
Max Consensus	89.8	45.9	82.0	29.0	96.0	38.0	93.0	34.5
Tool Avg.	64.7 ± 24	35.1 ± 24	63.7 ± 20	35.1 ± 25	75.7 ± 24	37.2 ± 25	73.3 ± 22	36.6 ± 21
ScType	66.5	38.5	66.5	43.5	85.7	36.0	85.7	47.1
scSorter	81.9	46.6	68.3	25.9	95.7	70.0	84.1	33.3
SCINA	25.6	2.0	33.9	3.6	34.9	0.0	34.8	1.1
SingleR	88.3	64.9	88.3	71.5	87.3	40.0	87.3	52.9
scPred	61.5	23.6	61.5	31.1	74.8	40.0	74.8	48.3

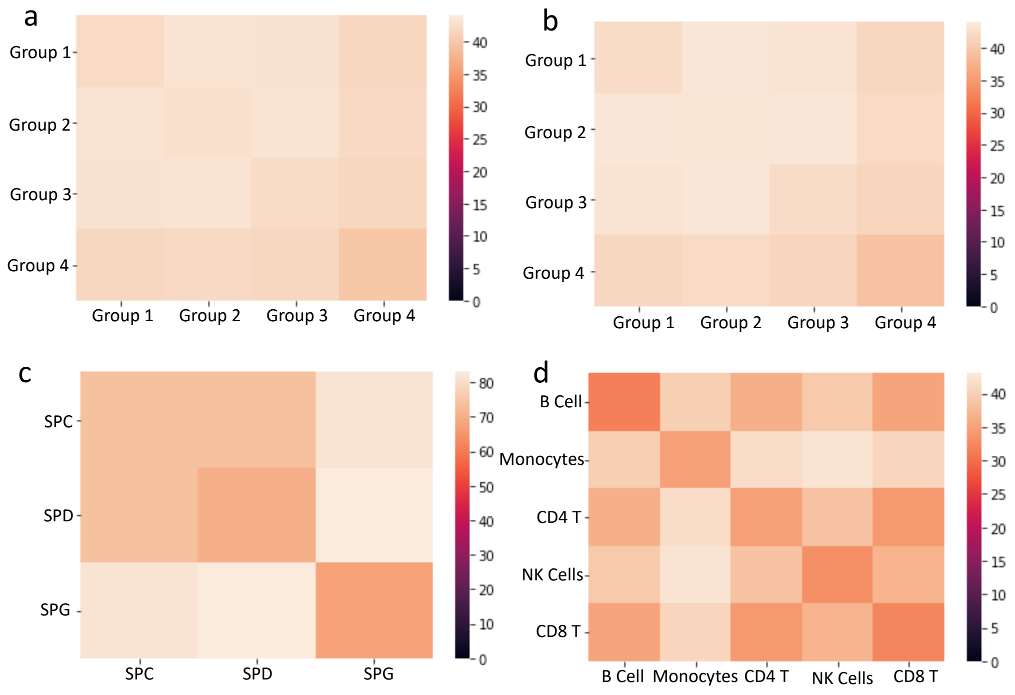

Fig. 5. Heat maps showing the average distance between cell type clusters in each data set. a. Simulation 0.7 b. Simulation 0.8 c. Testis d. PBMC

Table 3. Change between accuracy score before and after changing the number of markers used for each cell type in the Simulation 0.7 data sets. Accuracy difference is recorded for each cell type: Group 1 (1), Group 2 (2), Group 3 (3), and Group 4 (4). Group 1 had only two markers, Group 2 had only three markers, Group 3 had only four markers, and Group 4 had all five original marker genes.

Method	1	2	3	4
ScType	0.00	0.00	0.00	0.00
scSorter	-49.82	-6.34	-7.31	15.0
SCINA	0.00	36.65	0.39	0.42

Table 4. Change between accuracy score before and after changing the number of markers used for each cell type in the Simulation 0.8 data sets. Accuracy difference is recorded for each cell type: Group 1 (1), Group 2 (2), Group 3 (3), and Group 4 (4). Group 1 had only two markers, Group 2 had only three markers, Group 3 had only four markers, and Group 4 had all five original marker genes.

Method	1	2	3	4
ScType	0.00	0.00	0.00	0.00
scSorter	-23.46	-23.98	0.00	0.84
SCINA	-1.09	-0.45	-1.54	2.91

Table 5. Top 10 highly attributed DeepLIFT genes and top 10 positively differentially expressed (DE) genes for Spermatogonia (SPG), Spermatocytes (SPC), and Spermatids (SPD).

	SPG		SPC		SPD	
Rank	DeepLIFT	DE	DeepLIFT	DE	DeepLIFT	DE
1	mt-Rnr2	Ncl	Ldhc	Rsph1	Tnp1	Smcp
2	Ncl	Hsp90ab1	Ubb	Ldhc	Smcp	Tnp1
3	Hsp90ab1	Dazl	Fabp9	Lyar	Tsga8	Gm9999
4	Dazl	Anp32b	Pabpc1	Pabpc1	Dbil5	Fam229a
5	Prrc2c	Prrc2c	Meig1	Morf4l1	Gm9999	Dbil5
6	Tpr	Ssb	Calm2	Cox8c	Acrv1	Spata3
7	Anp32b	Taf7l	Morf4l1	Ccdc39	Odf2	DB30044l16Rik
8	mt-Nd1	mt-Co1	Calm1	Calm1	D830044l16Rik	Spata19
9	Rpl4	Rpl4	Tuba3b	Cdc42ep3	Ccdc136	X1700029H14Rik
10	Rbm39	Tpr	Rsph1	Meig1	mt-Rnr2	X2610318N02Rik

Table 6. Top 10 highly attributed DeepLIFT genes and top 10 positively differentially expressed (DE) genes for B Cells, Monocytes, Natural Killer Cells (NK Cells), CD4 T Cells (CD4), and CD8 T Cells (CD8).

	B Cells		Monocytes		NK Cells		CD4		CD8	
Rank	DeepLIFT	DE	DeepLIFT	DE	DeepLIFT	DE	DeepLIFT	DE	DeepLIFT	DE
1	CD74	HLA-DRA	FTH1	S100A8	GNLY	GNLY	LTB	IL32	CD8B	CD8B
2	HLA-DRA	CD74	TYROBP	S100A9	NKG7	NKG7	FTH1	AQP3	CD3E	CD8A
3	CD79A	CD79A	S100A9	CST3	TYROBP	GZMB	CD3E	CD3E	CTSW	RP11-291B21-2
4	CD79B	CD79B	S100A8	LYZ	RPS21	CLIC3	MT-CO2	LDHB	TPT1	S100B
5	HLA-DRB1	HLA-DPA1	FTL	FCN1	GZMB	FGFBP2	RPL34	MAL	EEF1A1	CCL5
6	LTB	HLA-DPB1	CST3	AIF1	RPL38	GZMA	S100A4	CORO1B	HLA-C	GZMK
7	HLA-DPA1	IGLL5	LYZ	LST1	HCST	CST7	CD3D	JUNB	CCL5	CARS
8	HLA-DPB1	HLA-DRB1	S100A4	TYMP	FCER1G	FCER1G	RPL37	CD27	MALAT1	CTSW
9	HLA-DRB5	MS4A1	HLA-DRB1	TYROBP	FTL	KLRB1	RPL7	CD3D1	CD3D	CPA5
10	CD37	HLA-DQA1	UBC	CFD	RPS26	PRF1	UBC	IL7R	EEF1D	NELL2
