# Supplementary figures and images for: Consensus label propagation with graph convolutional networks for single-cell RNA sequencing cell type annotation
Source: Bioinformatics. 2023 Jun 2;39(6):btad360. doi: 10.1093/bioinformatics/btad360 (PMC10272704; doi:10.1093/bioinformatics/btad360)

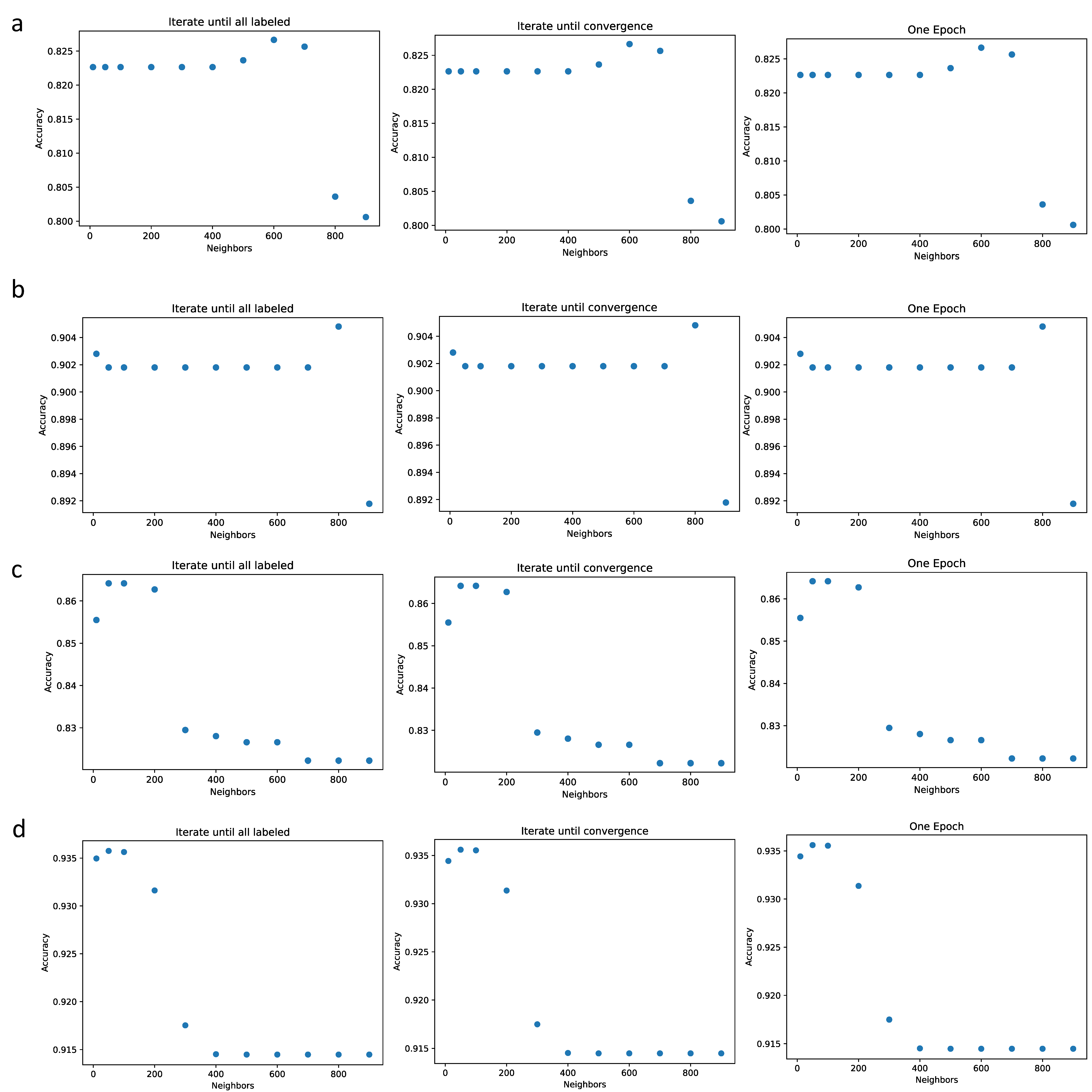

Supplement: btad360_Supplementary_Data [file btad360_supplementary_data.zip › knn_figure.tiff]

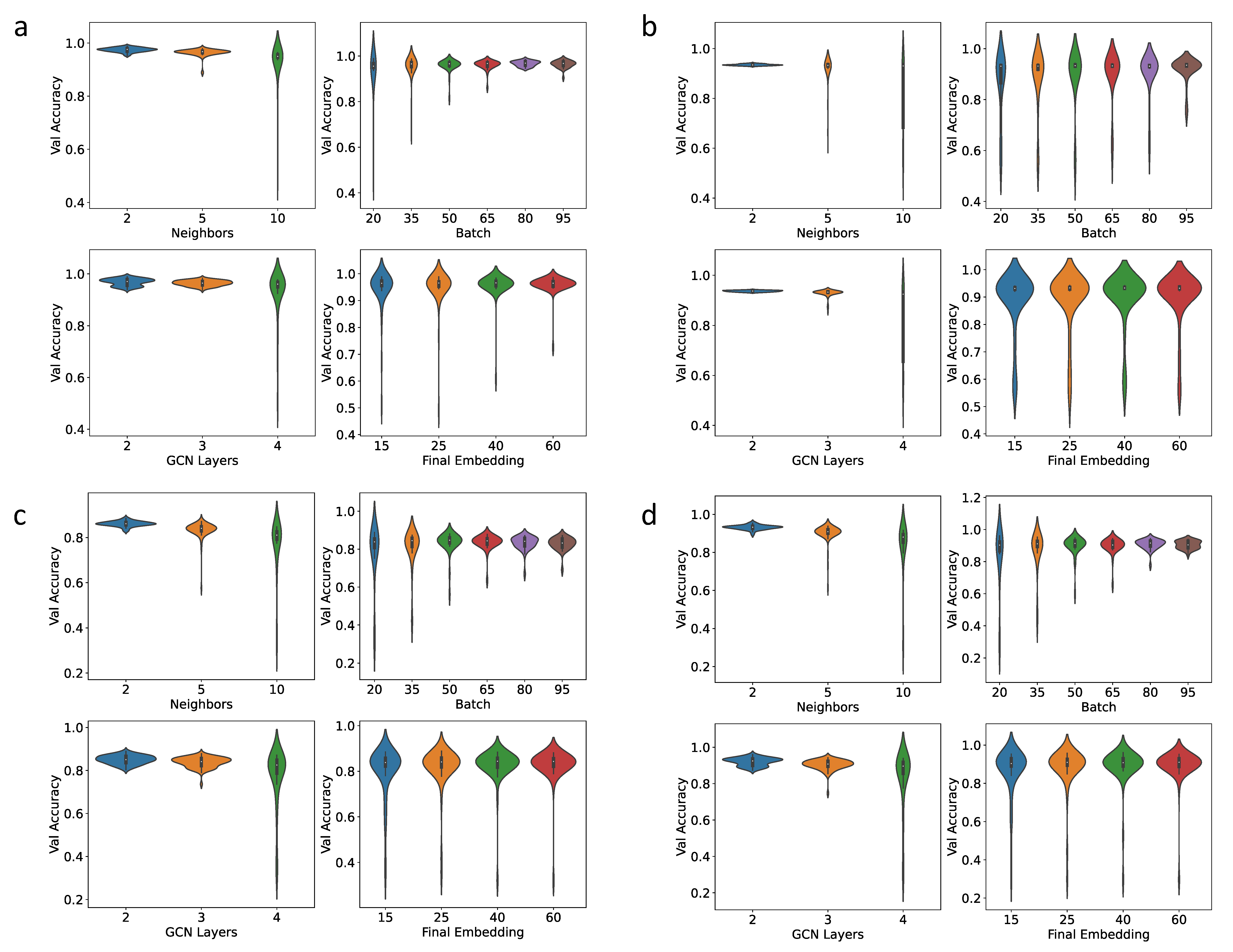

Supplement: btad360_Supplementary_Data [file btad360_supplementary_data.zip › param_figure.tiff]

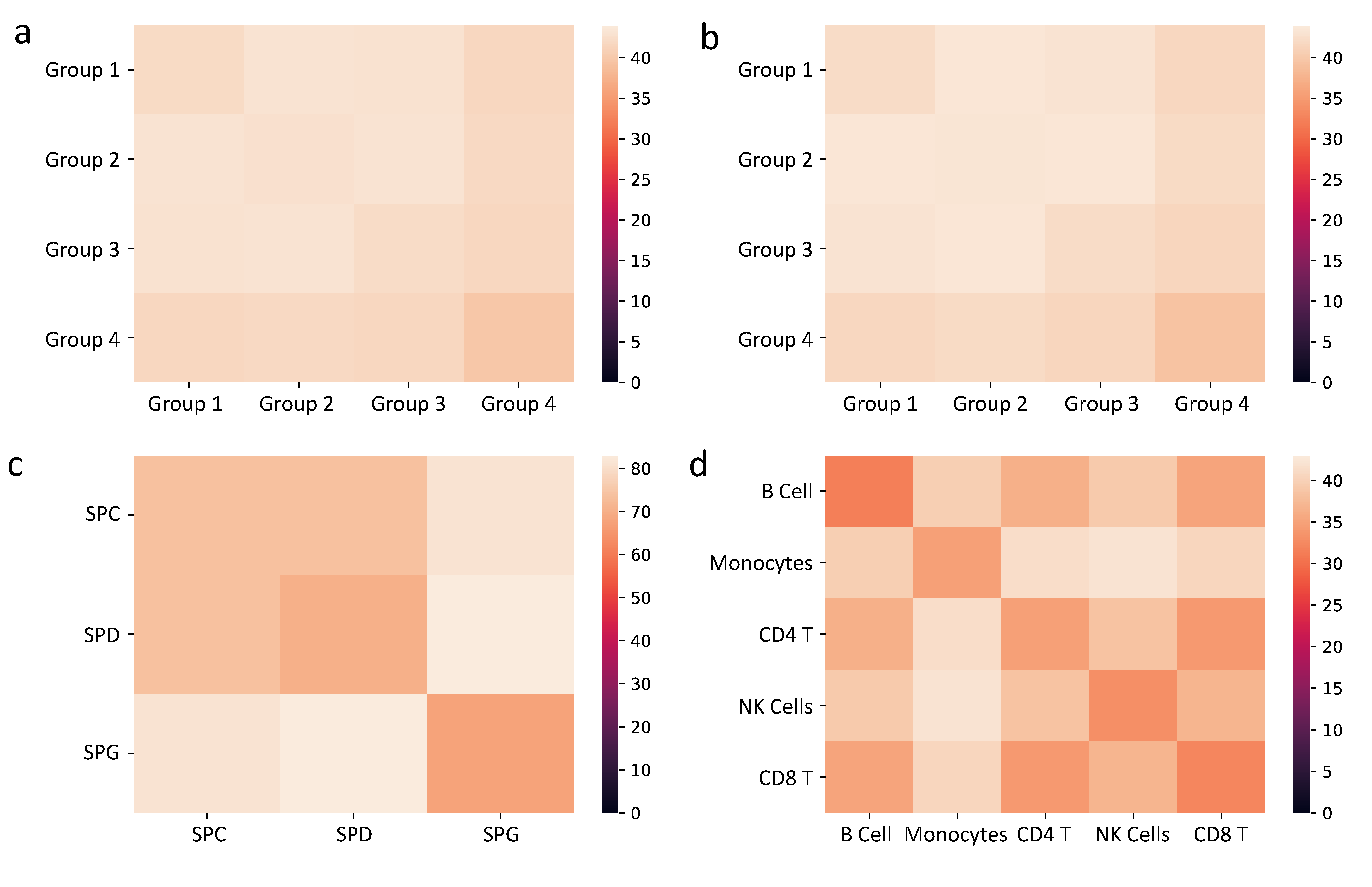

Supplement: btad360_Supplementary_Data [file btad360_supplementary_data.zip › cluster_similarity.tiff]

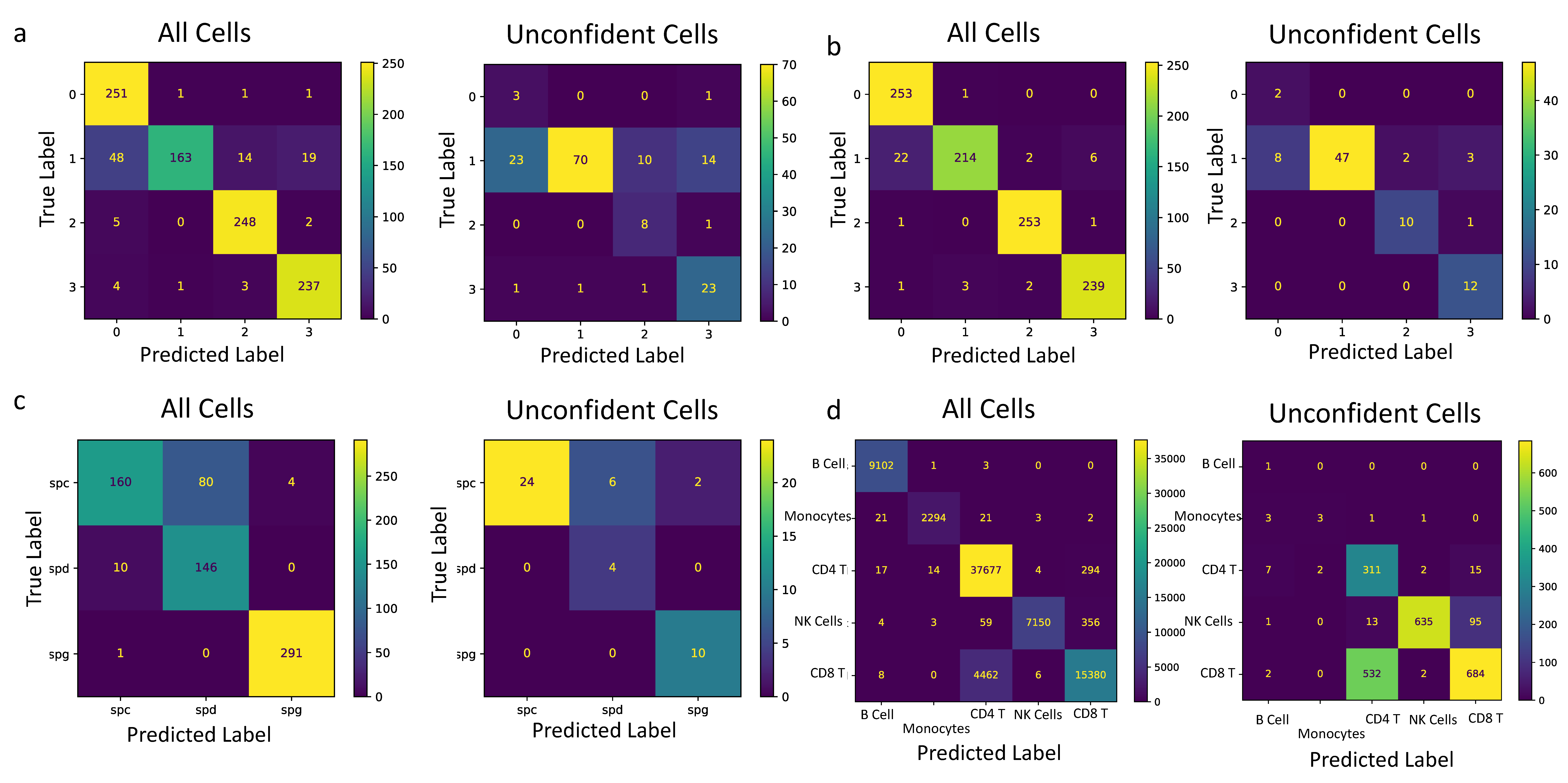

Supplement: btad360_Supplementary_Data [file btad360_supplementary_data.zip › cm_figure.tiff]
